# Rand Doctor's Distressing Death

**Published:** 1920-03-20

**Authors:** 


### Rand Doctor's Distressing Death.

Thousands of soldiers who have been in the *Ebani* hospital ship will be grieved at the recent death at Johannesburg of Lieut.-Col. D. Macaulay, M.A., M.B., C.M. (Edin.), who was crushed to death under his own motor-car after having left a dinner-party given by the Governor-General (Lord Buxton) at Bedford Farm.

Colonel Macaulay's car had not been running well, and the presumption is that the engine failed, and that when he left it the car was still in gear. When the engine was restarted the car bounded forward, knocked him down, and pinned him underneath.

The deceased went to Johannesburg many years ago and practised at the mines. He assisted in the research undertaken in order to minimise phthisis, and was popular with the mine workers. Colonel Macaulay served on the hospital ship plying with sick and wounded between German S.W. Africa and the Cape during General Botha's campaign. He served in the Mediterranean, and was given charge of the *Ebani* hospital ship in German East Africa. He sat in the Union Parliament as member for Denver, and had previously represented the same Rand constituency in the first Transvaal Parliament.

